# 应用二代测序技术研究慢性淋巴细胞白血病患者的免疫球蛋白可变区特征及预后价值

**DOI:** 10.3760/cma.j.cn121090-20240819-00310

**Published:** 2025-03

**Authors:** 祯 郭, 慧敏 金, 彤璐 邱, 莉颖 朱, 雨洁 吴, 海荣 仇, 琰 王, 祎 缪, 晖 金, 磊 范, 建勇 李, 奕 夏, 纯 乔

**Affiliations:** 南京医科大学第一附属医院 江苏省人民医院血液科淋巴瘤中心，南京 210029 Lymphoma Center, Department of Hematology, the First Affiliated Hospital of Nanjing Medical University, Jiangsu Province Hospital, Nanjing 210029, China

**Keywords:** 白血病，淋巴细胞，慢性，B细胞, 免疫球蛋白, 二代测序, 亚克隆, IGLV3-21, Leukemia, lymphocytic, chronic, B-cell, Immunoglobulin, Next-generation sequencing, Subclone, IGLV3-21

## Abstract

**目的:**

利用二代测序（NGS）技术分析慢性淋巴细胞白血病（CLL）患者免疫球蛋白（IG）重链和轻链可变区基因特征、亚克隆情况及预后价值。

**方法:**

采集2018年12月到2023年5月在江苏省人民医院初诊的36例CLL患者［包括12例Ｂ细胞受体（BCR）同型模式患者］的血液和（或）骨髓样本，应用NGS技术检测免疫球蛋白重链（IGH）、轻链（IGK、IGL）基因重排，研究IG基因在CLL中的特点及预后价值。

**结果:**

NGS检测IG重链可变区（IGHV）与Sanger测序具有明显的相关性和较好的一致性（*r*＝0.957，*P*<0.001）。36例患者中，9例（25.0％）患者IGHV有突变、27例（75.0％）患者IGHV无突变。MYD88突变在IGHV有突变患者中发生率更高［1/27（3.7％）对4/9（44.4％），*P*＝0.009］。BCR同型模式IGHV #8/8B亚组患者的12号染色体三体（+12）［4/11（36.4％）对1/25（4.0％），*P*＝0.023］的发生率更高，并更易发生Richter转化［8/11（72.7％）对4/25（16.0％），*P*＝0.002］。36例患者使用IGKV（36/36，100％）；15例使用IGLV（15/36，41.7％），其中IGLV3-21（5/15，33.3％）的使用率最高：患者均为Binet C分期及Rai Ⅲ～Ⅳ期，del（13）（q14）的发生率为60.0％（3/5）。使用和非使用IGLV3-21基因片段患者的中位至首次治疗时间（TTFT）分别为5.2（1.1～41.5）个月和9.9（0.1～94.4）个月。当定义2.5％的总检测片段条数（total reads）为IGHV亚克隆阈值时，检测出4例（4/36，11.1％）样本具有2个功能性克隆。有突变单克隆组（8/36，22.2％）和有突变双克隆组（1/36，2.8％）患者的中位TTFT分别为2.8（0.9～72.7）个月和12.8个月，中位总生存（OS）期分别为57.5（32.0～120.7）个月和51.8个月；无突变单克隆组（24/36，66.7％）和无突变双克隆组（2/36，5.6％）患者的中位TTFT分别为10.9（0.3～94.4）个月和6.3（0.1～12.5）个月，中位OS期分别为49.9（22.2～211.1）个月和30.0（9.6～50.3）个月（*P*>0.05）。

**结论:**

应用NGS技术检测IG基因重排，不仅能分析IGHV的突变状态、优势克隆、判断预后，还能够研究IGK/IGL的基因重排片段及亚克隆的使用情况，为IGLV3-21 CLL的不良预后研究提供了线索。IGHV无突变的双克隆可能提示预后不良。

## Abstract (en)

**Objective:**

To elucidate the genomic characteristics of the immunoglobulin（IG）heavy-chain variable region and light-chain variable region, the expression of subclones, and the prognostic significance in patients with CLL.

**Methods:**

Blood and/or bone marrow specimens were gathered from a cohort of 36 patients with CLL diagnosed at Jiangsu Province Hospital from December 2018 to May 2023, including 12 cases of B cell receptor（BCR）stereotyped patients. IG heavy-chain（IGH）and light-chain（IG Kappa [IGK] and IG lambda [IGL]）gene rearrangements were performed using next-generation sequencing（NGS）technology to analyze the characteristics and prognostic value in CLL.

**Results:**

NGS detection of IG variable region（IGHV）demonstrated a significant correlation and superior consistency with Sanger sequencing（*r*＝0.957, *P*<0.001）. Among the 36 patients, the IGH variant（IGHV）was observed in 9（25.0％）but not in 27（75.0％）participants. The incidence of the MYD88 mutation was higher among patients with mutated IGHV［1/27（3.7％）*vs* 4/9（44.4％）, *P*＝0.00］. A high incidence of trisomy 12 was observed in the IGHV #8/#8B subset［4/11（36.4％）*vs* 1/25（4.0％）, *P*＝0.023］, which were more likely to develop Richter transformation［8/11（72.7％）*vs* 4/25（16.0％）, *P*＝0.002］. In the patient cohort, 36 individuals（36/36, 100.0％）used the IGK variable, whereas 15 individuals（15/36, 41.7％）employed the IGL variable（IGLV）. IGLV3-21 reported the highest utilization rate in IGLV（5/15, 33.3％）. Remarkably, patients with CLL with IGLV3-21 fragments were exclusively observed in the Binet C stage and Rai Phase Ⅲ–Ⅳ, with an incidence of del（13）（q14）at 60.0％（3/5）. The median time to first treatment（TTFT）of patients with or without IGLV3-21 fragments was 5.2（1.1–41.5）and 9.9（0.1–94.4）months, respectively. Using the total reads threshold of 2.5％, 4（4/36, 11.1％）samples were detected to have two IGHV productive clones. The median TTFT and overall survival（OS）time were 2.8（0.9–72.7）and 12.8 months in patients with one mutated clone and 57.5（32.0–120.7）and 51.8 months in those with two mutated clones, respectively. The median TTFT and OS time were 10.9（0.3–94.4）and 6.3（0.1–12.5）months in patients with one unmutated clone and 49.9（22.2–211.1）and 30.0（9.6–50.3）months in those with multiple unmutated clones, respectively（*P*>0.05）.

**Conclusion:**

Detection of IG gene rearrangements using NGS technology not only facilitates the analysis of the IGHV mutation status, dominant clones, and prognostic value but also contributes to the exploration of IGK/IGL gene rearrangement fragments and the utilization of subclones. Further, it provides information about the poor prognosis of IGLV3-21 CLL. The shortened survival of the two unmutated clone groups in the IGHV unmutated group may indicate a poor prognosis.

慢性淋巴细胞白血病（CLL）是西方白种人中最常见的白血病，发病率逐年上升[1]，与西方相比，中国的发病率仅为西方的1/10[2]。CLL是一种以成熟B淋巴细胞在骨髓、淋巴组织和血液中聚集为特点的血液系统恶性肿瘤，临床病程和预后呈现高度异质性，具有独特的细胞形态、免疫表型、细胞遗传学及分子生物学特征。B细胞受体（BCR）对B细胞的生长发育至关重要，免疫球蛋白（IG）是BCR的主要组成部分。可变区（多样性区）连接区［V（D）J］重排、体细胞超突变（SHM）和类别转换重组（CSR）会导致IG位点的平衡易位，IG基因片段的偏好使用特征可能参与CLL的发生和发展，为预后判断提供依据。IG重链可变区（IGHV）基因突变状态是CLL的重要预后因素[3]。国际上公认IGHV无突变的CLL患者疾病进展更快，预后更差[4]，但对于CLL患者IG轻链（IGK、IGL）的使用情况、亚克隆表达情况及其预后价值还处于探索阶段。本研究应用二代测序（NGS）技术分析了36例CLL患者的IG（IGH、IGK、IGL）基因片段使用情况及亚克隆信息，探索克隆性重排在CLL中的应用价值，为患者的预后及治疗选择提供进一步指导。

## 病例与方法

1. 病例：本回顾性研究共纳入2018年1月至2023年5月在南京医科大学第一附属医院血液科就诊的36例初诊CLL患者（包括12例BCR同型模式患者，其中#8亚组5例、#8B亚组6例、#28A亚组1例）。诊断符合国际CLL工作组2018（IWCLL 2018）诊断标准，在患者首次入院后24 h内采集临床数据，包括性别、年龄、WBC、淋巴细胞绝对计数（ALC）、HGB、PLT、LDH、β_2_微球蛋白。患者中位年龄53（22～83）岁，其中男25例，女11例。根据Rai分期，低危组（0期）患者0例、中危期（Ⅰ～Ⅱ期）患者11例、高危组（Ⅲ～Ⅳ期）患者25例。根据Binet分期，A期患者0例、B期患者13例、C期患者23例。所有患者随访截止日期为2023年11月。

2. IG基因NGS技术检测：使用QIAmpDNA试剂盒（德国Qiagen公司产品）提取基因组DNA，使用LymphoTrack Assay-Miseq试剂盒对样本进行PCR扩增后对扩增产物进行文库纯化（AMPure XP beads试剂盒，美国赛默飞世尔科技公司产品）。产物纯化后使用KAPA Library Quantification Kit（Illumina平台）进行文库浓度定量，使用Miseq测序仪（北京旌准医疗科技有限公司产品）进行测序。根据国际ImMunoGeneTics协会定义标注可变区互补性决定区域3（CDR3）区序列，同时鉴定各序列相应的V、D、J基因构成。测序结果应用IMGT/V-QUEST数据库与已知参考序列进行比对分析（http://imgt.cines.fr）[5]，与胚系IGHV序列相比，相似性<98％的IGHV序列被定义为IGHV有突变，相似性≥98％被定义为IGHV无突变[6]–[8]。BCR同型模式的纳入标准必须满足以下5点[9]：①相同的CDR3长度；②CDR3氨基酸一致性≥50％；③CDR3氨基酸相似性≥70％；④CDR3特定的氨基酸排列模式；⑤IGHV属于同一家族（clan）。

3. 细胞遗传学：采用FISH技术，分别使用GLP D13S319、LSI TP53探针、LSI ATM DNA探针、CEP+12 DNA探针（美国雅培分子公司）检测del（13）（q14）、del（17）（p13）、del（11）（q23）和+12等异常[10]，利用Olympus BX60荧光显微镜观察间期细胞荧光杂交信号，具体步骤详见说明书。del（13）（q14）、del（17）（p13）、del（11）（q23）和+12细胞遗传学异常分别以>10％、5％、8％、5％为阳性结果判断标准。

4. 靶向测序：收集患者初诊时外周血或骨髓单个核细胞5.0×10^6^个，采用QIAamp DNA blood mini kit试剂盒（德国Qiagen公司产品）提取基因组DNA，以至少100 ng DNA进行扩增建库，应用Illumina MiSeq测序仪测序，平均测序深度1000×，使用TMAP软件（美国赛默飞世尔科技公司产品）分析测序结果。根据文献报道选取突变发生率较高的4个基因进行研究[11]–[12]，分别是TP53、ATM、NOTCH1和MYD88。

5. IGHV的克隆多样性：NGS技术进行IGHV基因的检测，除了主克隆还能检测到不同的亚克隆。由于没有公认的标准定义亚克隆的阈值，本研究参考Stamatopoulos等[13]和Bourbon等[14]的研究方法，分别定义总检测片段条数（total reads）≥2.5％及≥5.0％为亚克隆的阈值进行分析。

6. 统计学处理：采用GraphPad Prism 8.0软件进行统计分析。计数资料以例数（％）表示，并使用*χ*^2^检验或Fisher确切概率法进行分析。连续变量以*x*±*s*表示，非正态分布的计量资料以*M*（范围）描述，并使用非配对*t*检验和Mann Whitney *U*检验分析。总生存（OS）期被定义为从诊断到死亡或最后一次随访的时间，至首次治疗时间（time to first treatment，TTFT）被定义为诊断至首次治疗的时间。相关性分析采用非参数Spearman检验分析。采用Bland-Altman分析方法评估两种方法之间的一致性。生存曲线由Kaplan-Meier方法构建，各组患者生存曲线比较使用Log-rank检验。*P*<0.05为差异有统计学意义。

## 结果

1. NGS技术和Sanger测序检测IGHV突变状态的方法比较：采用NGS技术检测患者的IGHV突变情况，同时与Sanger测序进行对比，发现两种方法在胚系基因的符合率方面呈明显正相关（*r*＝0.957，95％ *CI* 0.912～0.979，*P*<0.001；图1A）。Bland-Altman分析显示这两种方法具有较好的一致性（Bias＝0.003，95％一致性界限−0.313～0.319，*P*＝0.743；图1B）。两种方法所检测的样本中，均为27例（27/36，75.0％）无突变、9例（9/36，25.0％）有突变，未发现临界突变状态的样本（97％≤符合率<98％）。因此，两种方法在判断IGHV突变状态的结果方面无差异。

**图1:**
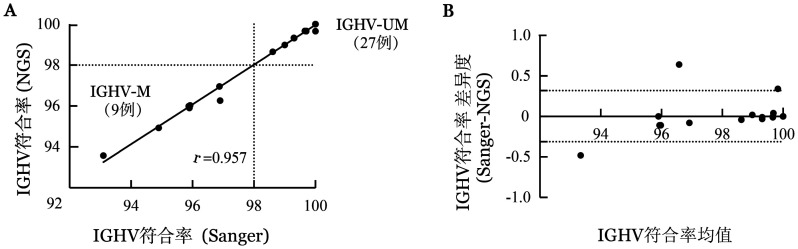
Sanger测序和二代测序（NGS）技术检测慢性淋巴细胞白血病患者IGHV突变情况的比较 **A** 两种测序技术检测IGHV基因符合率的相关性分析；**B** Bland-Altman方法比较两种技术检测IGHV基因符合率的一致性 **注** IGHV：免疫球蛋白重链可变区；IGHV-M：IGHV有突变；IGHV-UM：IGHV无突变

2. IGHV突变状态及IG重链使用片段的分析：本研究中有14例患者（14/36，38.9％）IGHV基因序列与胚系基因完全一致，均为IGHV无突变。IGHV4基因家族的使用率（16/36，44.4％）最高，主要使用片段为IGHV4-39（12/16，75.0％）和IGHV4-34（3/16，18.8％），其次为IGHV3基因家族（14/36，38.9％），主要使用片段为IGHV3-7（3/14，21.4％）及IGHV3-9（3/14，21.4％）（图2A）。IGHD中IGHD6（15/36，41.7％）和IGHD3（12/36，33.3％）家族使用率较高（图2B）。IGHJ中IGHJ5家族（14/36，38.9％）的使用率最高，其次为IGHJ4家族（11/36，30.6％）和IGHJ6家族（5/36，13.9％），其中最常见的基因片段为IGHJ 5*02，占38.9％（11/36）（图2C）。

**图2:**
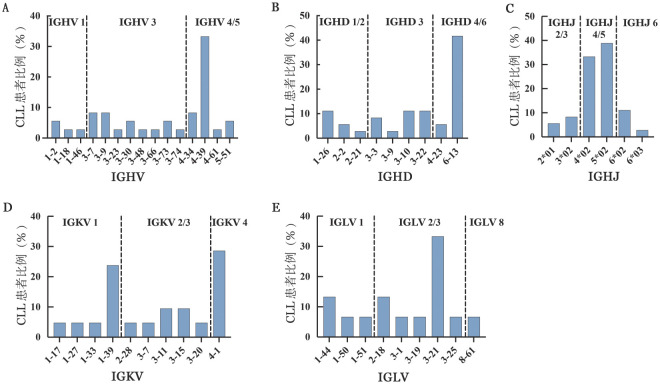
慢性淋巴细胞白血病（CLL）患者免疫球蛋白（IG）基因的主要使用片段 **A-C** CLL患者IGHV、IGHD、IGHJ的主要使用片段；**D** CLL患者IGKV的主要使用片段；**E** CLL患者IGLV的主要使用片段 **注** IGH：IG重链；IGH、IGL：IG轻链；V-D-J：可变区-多样性区-连接区

3. IGHV突变状态与临床特征及预后价值：如表1所示，从临床特征方面进行分析发现，IGHV有突变患者均属于Rai Ⅲ～Ⅳ期（*P*＝0.034）。IGHV有突变患者的ALC明显高于无突变患者（*P*＝0.036）。+12、del（13）（q14）、del（11）（q22）和del（17）（p13）在IGHV无突变和有突变患者中的发生率差异均无统计学意义（均*P*>0.05）。IGHV有突变患者MYD88突变发生率更高［1/27（3.7％）对4/9（44.4％），*P*＝0.009］。IGHV突变状态与患者临床特征，包括年龄、性别、WBC、HGB、PLT、LDH、β_2_微球蛋白等差异均无统计学意义（均*P*>0.05）。IGHV无突变的患者更易发生Richter转化［12/27（44.4％）对0/9（0），*P*＝0.016］，IGHV无突变和有突变患者的中位OS期分别为49.3（5.6～211.1）个月和56.9（32.0～120.7）个月（*P*>0.05）。

**表1:** IGHV-M和IGHV-UM的慢性淋巴细胞白血病患者临床资料比较

临床特征	IGHV-M（9例）	IGHV-UM（27例）	统计量	*P*值
年龄［岁，*x*±s］	49.84±9.69	54.23±11.47	1.061	0.296
性别［例（％）］			0.044	0.834
男	6（66.7）	19（70.4）		
女	3（33.3）	8（29.6）		
Rai分期［例（％）］			5.280	0.034
Ⅰ～Ⅱ期	0（0）	11（40.7）		
Ⅲ～Ⅳ期	9（100）	16（59.3）		
Binet分期［例（％）］			3.251	0.114
B期	1（11.1）	12（44.4）		
C期	8（88.9）	15（55.6）		
WBC［×10^9^/L，*M*（范围）］	88.65（4.74～113.25）	44.79（4.75～246.54）	1.599	0.120
ALC［×10^9^/L，*M*（范围）］	70.80（3.60～297.00）	9.18（2.10～36.17）	1.975	0.036
HGB［g/L，*M*（范围）］	109.63（86.00～154.00）	109.90（36.00～163.00）	0.053	0.958
PLT［×10^9^/L，*M*（范围）］	115.50（46.00～174.00）	114.00（22.00～321.00）	0.827	0.414
LDH［U/L，*M*（范围）］	236.44（151.00～503.00）	325.90（132.00～990.00）	1.244	0.246
β_2_-MG［mg/L，*M*（范围）］	4.69（1.73～7.06）	24.38（1.80～398.50）	0.664	0.241
FISH检测结果［例（％）］				
del（11）（q22）	0（0）	2（7.4）	0.706	0.401
del（17）（p13）	1（11.1）	3（11.1）	0.000	>0.999
del（13）（q14）	3（33.3）	5（18.5）	0.857	0.384
+12	0（0）	5（18.5）	1.935	0.302
基因突变［例（％）］				
ATM	0（0）	9（33.3）	4.000	0.076
TP53	1（11.1）	5（18.5）	0.267	0.606
NOTCH1	0（0）	8（29.6）	3.429	0.160
MYD88	4（44.4）	1（3.7）	9.368	0.009

**注** IGHV：免疫球蛋白重链可变区；IGHV-M：IGHV有突变；IGHV-UM：IGHV无突变；ALC：淋巴细胞绝对计数；β_2_-MG：β_2_微球蛋白；+12：12号染色体三体

4. BCR同型模式分析：本研究中患者的IG重链3号互补决定区（HCDR3）的中位氨基酸长度为17（9～23）个。IGHV无突变患者BCR同型模式的概率高于IGHV有突变的患者［12/27（44.4％）对0/9（0），*P*>0.05］。12例BCR同型模式（12/36，33.3％）患者中，#8亚组有5例（5/36，13.9％）、#8B亚组有6例（6/36，16.7％）、#28A亚组有1例（1/36，2.8％）。#8/8B亚组患者+12［4/11（36.4％）对1/25（4.0％），*P*＝0.023］的发生率更高，非#8/8B亚组患者的del（13）（q14）［0/11（0）对7/25（28.0％），*P*>0.05］、del（11）（q22）［0/11（0）对2/25（8.0％），*P*>0.05］和del（17）（p13）［0/11（0）对4/25（16.0％），*P*>0.05］的发生率更高，但差异均无统计学意义。ATM突变、MYD88突变、TP53突变和NOTCH1突变在#8/8B亚组和非#8/8B亚组患者中的发生率分别为36.4％（4/11）和20.0％（5/25）、0（0/11）和20.0％（5/25）、9.1％（1/11）和20.0％（5/25）、27.3％（3/11）和20.0％（5/25）（*P*>0.05）。#8/8B亚组更易发生Richter转化［8/11（72.7％）对4/25（16.0％），*P*＝0.002］，且中位TTFT［5.6（0.2～94.4）个月对9.9（0.3～72.8）个月，*P*＝0.034］和中位OS期［30.6（5.6～211.1）个月对56.9（32.0～120.7）个月，*P*＝0.006］较非#8/8B亚组患者显著缩短，预后更差。

5. IGK和IGL的基因片段使用特征：36例患者中，36例使用IGKV（36/36，100％），15例使用IGLV（15/36，41.7％）。IGKV1（11/36，30.6％）、IGKV3（11/36，30.6％）和IGKV4（7/36，19.4％）家族最常被使用，其中IGKV4-1（7/36，19.4％）和IGKV1-39（6/36，16.7％）使用率最高（图2D）。使用IGKV4-1基因片段的CLL患者的中位TTFT和OS期分别为2.7（0.90～17.0）个月和42.9（30.6～66.0）个月，而非使用IGKV4-1基因片段的患者中位TTFT和OS期分别为9.9（0.2～94.4）个月和51.8（5.6～211.1）个月（*P*>0.05），Richter转化发生率分别为42.9％（3/7）和31.0％（9/29）。使用IGKV1-39基因的患者均属于BCR同型模式#8/8B，中位TTFT和OS期分别为19.0（0.1～94.4）个月和55.9（5.6～211.1）个月，而非使用IGKV1-39基因片段的患者中位TTFT和OS期分别为15.0（0.3～72.7）个月和56.8（23.8～120.7）个月（*P*>0.05）。最常被使用的IGLV家族是IGLV3（8/15，53.3％）和IGLV1（4/15，26.7％），其中IGLV3-21（5/15，33.3％）的使用率最高（图2E），患者均属于Binet C分期及Rai Ⅲ～Ⅳ期。使用和非使用IGLV3-21基因片段患者的del（13）（q14）的发生率分别为60.0％（3/5）和16.1％（5/31），中位TTFT分别为5.2（1.1～41.5）个月和9.9（0.1～94.4）个月（*P*>0.05）。

若只考虑功能性克隆，以2.5％为阈值，4例（4/36，11.1％）样本具有2个功能性克隆，其中1例样本（样本号5）的双克隆具有不同的V-D-J重排，表现不一致的突变状态。另外3例样本中，双克隆具有一致的突变状态（无突变2例、有突变1例），图3A展示了样本的亚克隆及V区基因使用情况。即使双克隆具有相同的V-D-J重排，但核苷酸序列仍会有不同，证明了样本具有克隆内多样性。以5.0％为阈值，1例（1/36，2.8％）样本具有2个功能性克隆，为无突变状态（样本号55，图3B）。

**图3:**
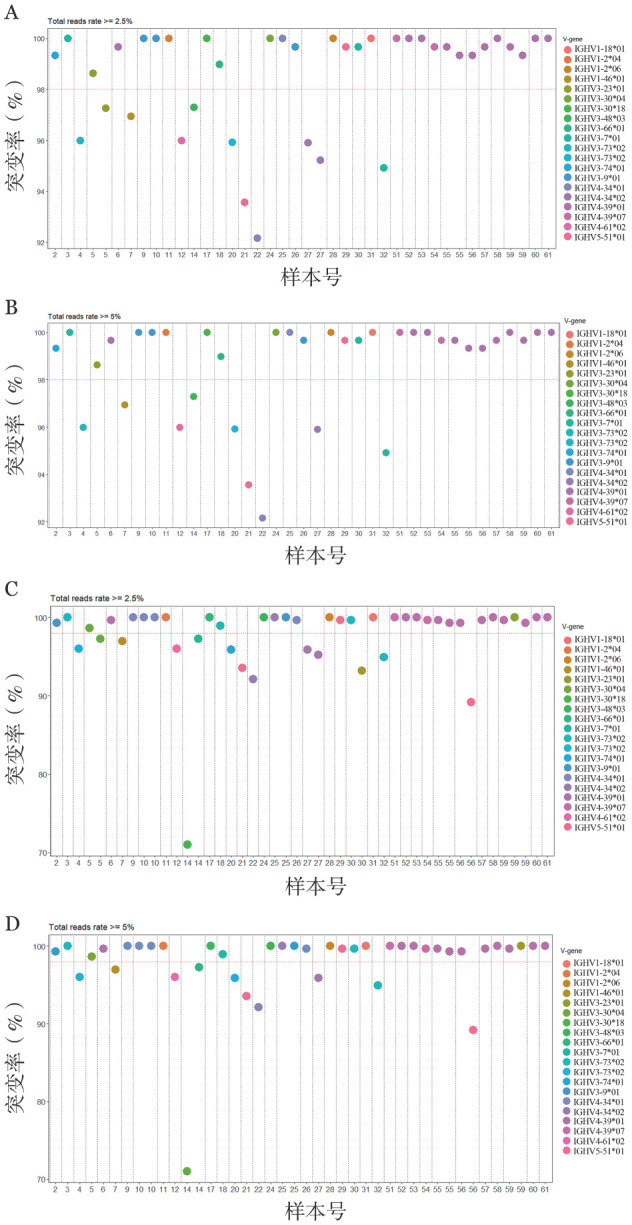
慢性淋巴细胞白血病患者主克隆及亚克隆免疫球蛋白重链可变区（IGHV）基因使用情况 **A** 仅考虑功能性克隆且亚克隆的阈值为2.5％，样本IGHV基因使用情况；**B** 仅考虑功能性克隆且亚克隆的阈值为5.0％，样本IGHV基因使用情况；**C** 同时考虑功能性和非功能性克隆且亚克隆的阈值为2.5％，样本IGHV基因使用情况；**D** 同时考虑功能性和非功能性克隆且亚克隆的阈值为5.0％，样本IGHV基因使用情况

因此根据克隆的数量及突变状态，当以2.5％为阈值时，患者可划分成5组：有突变单克隆组（8/36，22.2％）、有突变双克隆组（1/36，2.8％）、无突变和有突变双克隆组（1/36，2.8％）、无突变单克隆组（24/36，66.7％）、无突变双克隆组（2/36，5.6％）。有突变单克隆组和有突变双克隆组患者的中位TTFT分别为2.8（0.9～72.7）个月和12.8个月，中位OS期分别为57.5（32.0～120.7）个月和51.8个月。无突变单克隆组和无突变双克隆组患者的中位TTFT分别为10.9（0.3～94.4）个月和6.3（0.1～12.5）个月，中位OS期分别为49.9（22.2～211.1）个月和30.0（9.6～50.3）个月。在五组中，无突变和有突变双克隆组患者（例5）的中位TTFT（1.1个月）最短，中位OS期为49.3个月。

当以5.0％为阈值时，患者可划分成3组：有突变单克隆组（9/36，25.0％）、无突变单克隆组（26/36，72.2％）、无突变双克隆组（1/36，2.8％）。三组患者的中位TTFT分别为3.3（0.9～72.7）个月、7.3（0.1～94.4）个月和12.5个月，中位OS期分别为56.9（32.0～120.7）个月、49.3（5.6～211.1）个月和50.3个月。

若纳入非功能性克隆进行分析，以2.5％为阈值，9例（9/36，25.0％）样本具有2个及以上克隆，其中3例样本（样本号5、30、56）的多个克隆具有不同的V-D-J重排，且表现不一致的突变状态。另外6例样本中，多个克隆具有一致的突变状态（无突变4例，突变2例），图3C展示了样本的亚克隆及V区基因使用情况。以5.0％为阈值，6例（6/36，16.7％）样本具有1个以上克隆，其中1例样本（样本号56）的多个克隆表现不一致的突变状态。另外5例样本中，两个克隆具有一致的突变状态（无突变4例，突变1例）（图3D）。

## 讨论

本研究利用高通量NGS技术对初诊CLL患者IG基因的重链和轻链进行测序，不仅分析了IGHV-IGHD-IGHJ基因片段使用特征，还分析了患者的IGK/IGL基因使用情况，同时分析了患者样本中的克隆多样性，探索了NGS技术在检测CLL患者IG基因使用情况及预后价值方面的特点及优势。

与Sanger测序相比，两种方法检测出的胚系基因符合率具有高度一致性，并且都检测出了符合IGHV同型模式的克隆序列。约10％的CLL患者发生多次重排或存在克隆内多样性，由于克隆频率较低或扩增效率低，Sanger测序难以检出此类克隆[15]，而NGS技术可以检测同一患者中存在的次要相关克隆（克隆内多样性产生的亚克隆）和不相关的克隆（不同的亚克隆）[16]，因此NGS技术较Sanger测序的灵敏度、特异性均有所提升。本研究中，IGHV有突变患者9例（9/36，25.0％），进一步比较VH基因的家族特点发现IGHV3和IGHV4家族使用率较高，而IGHV1的使用率则较低，其中IGHV4-39的使用率最高，其次是IGHV3-7、IGHV3-9和IGHV4-34，而IGHV1-69片段未被检测出，这与其他亚洲人群的IGHV研究结果一致[17]。

从对轻链的使用情况分析可以发现，58.3％的患者使用IGKV，41.7％的患者使用IGLV，家族使用率排序分别为IGKV1>IGKV3>IGKV4和IGLV3>IGLV1>IGLV2，这些顺序和正常B细胞中轻链家族的使用频率相似[18]。使用频率最高的IGLV基因片段是IGLV3-21，其中3例（60.0％）为IGHV无突变，2例（40.0％）为IGHV有突变。使用IGLV3-21基因片段的CLL患者均属于Binet C分期及Rai Ⅲ～Ⅳ期，del（13）（q14）的发生率更高。国外的研究报道携带IGLV3-21^R110^的CLL患者可以划分为与危险分层相关的独立亚组，该亚组患者侵袭性高且预后不良[19]。而在中国人群中，IGLV3-21能否也成为CLL患者预后的独立影响因素之一，未来还需要进一步扩大样本量进行验证。

IGHV基因突变状态是国际上判断CLL预后的金标准，据报道[20]–[21]，只有5％的CLL患者样本可以通过Sanger测序检测出双克隆重排，使用NGS技术可以有效提高亚克隆的检出率并观察到克隆多样性。为了排除正常反应性B细胞的影响，本研究分别选择total reads为2.5％和5.0％的临界值来定义亚克隆。以2.5％为阈值，检测出11.1％的患者携带两个功能性克隆，其中1例具有不同突变状态的2个克隆（例5）。欧洲CLL研究倡议组织（ERIC）对IG检测结果的最新解读显示[22]，当患者携带具有不同突变状态的两个克隆应判断为无突变状态，同时密切随访。本研究结果也显示，例5的生存情况较差。长期以来，人们认为UM-CLL（IGHV无突变的CLL）和M-CLL（IGHV有突变的CLL）来自B细胞的不同成熟阶段，经过或未经过生发中心，是两类独立的个体[23]，但随着越来越多与IGHV无关的亚克隆被发现，这种观点受到了挑战[24]。有学者认为，IGHV的突变状态可能是SHM过程中不同的DNA修复机制的结果，这取决于CLL细胞的增殖速率[25]，这可能也解释了在CLL患者中可以同时观察到有突变和无突变状态亚克隆。

Stamatopoulos等[13]根据NGS技术检测的亚克隆数量和突变状态定义了5个亚组，包括有突变多克隆组、有突变单克隆组、无突变和有突变多克隆组、无突变单克隆组（+VH3-21）和无突变多克隆组，对患者的预后进行精细化分层。无论是只分析功能性克隆，还是同时纳入功能性和非功能性克隆进行分析，与IGHV无突变单克隆组相比，无突变多克隆组患者生存期缩短，这与本研究的结果类似，后续将纳入更多样本进行研究。此外，新的NGS-IGHV分层在验证队列中得到验证和应用，无突变多克隆组患者的预后最差，从而指导了92例患者的预后评估和治疗决策[13]。

综上所述，本研究是在国内率先采用NGS进行IG基因可变区检测的研究，分析了CLL患者IG重链和轻链的使用特征、亚克隆情况和克隆多样性，提示IGHV无突变的双/多克隆可能对患者生存具有不良影响，并为IGLV3-21 CLL的不良预后提供了进一步研究的线索。未来希望通过扩大样本量并开展多中心联合研究，探索IGK/IGL基因片段如IGLV3-21^R110^在中国人群中的预后价值，应用NGS技术对患者进行精细化分层从而指导个体化和精准治疗。
